# Reducing the opioid overdose death toll in North America

**DOI:** 10.1371/journal.pmed.1002626

**Published:** 2018-07-31

**Authors:** Wayne D. Hall, Michael Farrell

**Affiliations:** 1 Centre for Youth Substance Abuse Research, the University of Queensland, St Lucia, Australia; 2 National Addiction Centre, Kings College London, London, United Kingdom; 3 National Drug and Alcohol Research Centre, the University of New South Wales, Sydney, Australia

## Abstract

In this Perspective, Wayne D. Hall & Michael Farrell discuss the current need for alternative strategies in the rising opioid crisis in the US and the necessity to fund evidence-based treatment initiatives to reduce the death toll.

Over the past two decades, the United States population has experienced an extraordinary increase in the rates of death from opioid overdose. A steep rise in fatal overdoses caused by pharmaceutical opioids observed over the last 15 years [1] has been overtaken by rapidly increasing rates of heroin and illicit fentanyl overdose death as the supply of prescription opioids has been reduced [2]. In 2017, there were estimated to be 68,400 drug overdose deaths in the US [3], more than the annual number of deaths from AIDS at the height of that epidemic. These deaths have contributed to an overall decrease in life expectancy among middle-aged white people in the US [4].

Research by Angela Russolillo and colleagues, published this week in *PLOS Medicine* [5], highlights an effective way of reducing these opioid overdose deaths, namely by increasing the number of opioid-dependent persons who receive methadone-assisted treatment. The authors studied mortality in a high-risk population of 14,530 opioid-dependent offenders in British Columbia, Canada who were treated and followed-up using death and treatment registers from 1998 to 2015. Their findings showed that receipt of methadone-assisted treatment in the community substantially reduced mortality from external causes of death (adjusted hazard ratio [AHR] = 0.41 [95% CI 0.33–0.51]) and nonexternal causes of death (AHR = 0.27 [95% CI 0.23–0.33]) as well as substantially reducing the risk of death from opioid overdose (AHR = 0.39 [95% CI 0.30–0.50]). These differences persisted after controlling for potential confounders and when a competing risks analysis was conducted.

Opioid-dependent offenders are a population at high risk of fatal opioid overdose for several reasons. They have a very elevated risk of overdosing when they leave prison with a reduced tolerance and return to opioid use, as most do without treatment [6]. The risk of dying accumulates with repeated cycles of incarceration and release. These offenders are also often of low socioeconomic status, have high rates of homelessness, and are less likely to seek, or receive, treatment for their opioid dependence. As Russolillo and colleagues show, despite these disadvantages, opioid-dependent offenders who were enrolled in methadone-assisted treatment had a much lower risk of dying from an opioid overdose.

There are additional good public health reasons for expanding methadone-assisted treatment for opioid dependence. Systematic reviews of clinical trials and observational studies show that methadone reduces the frequency of illicit opioid use, the criminal activity that users engage in to fund their opioid use, and their risk of rearrest [7]. It also reduces infection and transmission of blood-borne virus infections, such as hepatitis C and HIV [7].

US President Donald Trump has declared opioid overdose deaths a public health emergency and appointed a commission to advise him on how to end it [8]. The Commission’s report recommended (among other things) an expansion of medication-assisted treatment for opioid dependence, but the President has so far not allocated any funds to expand treatment. He has preferred publicly appealing, low-cost strategies, including media campaigns about the dangers of opioids, reducing opioid prescriptions, and capital punishment for opioid dealers [9]. The first of these—media campaigns—will do little on its own. Regarding the second, while reducing opioid prescriptions would be useful, this approach fails to address the increasing rates of death from heroin and illicit fentanyl. Finally, the third strategy has little chance of reducing opioid deaths.

A failure to expand effective treatment will ensure that opioid overdose deaths in the US continue to increase. This is especially likely if state governments adopt other policies that will increase the risk of fatal overdoses, e.g., imprisoning more opioid users, charging opioid users with murder if they supply drugs to peers who die of an overdose [10], and restricting the treatment offered to detoxification and drug-free rehabilitation [7].

If the US government wants to reduce the unconscionable toll that opioid overdose deaths are taking among its citizens, then it needs to adopt the effective public health approaches advocated by expert committees [11] and commissions [8]. This should include increasing access to methadone- and buprenorphine-assisted treatment [12] and maximising their uptake by funding educational programs to reduce the stigma of addiction that discourages treatment seeking. The latter will need to address the prejudice in the community, often shared by opioid-dependent people and treatment practitioners, that drug-free approaches to treatment are the only acceptable way to treat opioid dependence.

Expanded treatment will need to be accompanied by other measures to reduce overdose deaths among opioid users who are not in treatment. These should include the distribution of the opioid antagonist naloxone to first responders and people seeking treatment for opioid dependence so that bystanders can reverse opioid overdoses and prevent deaths [13]. It may also include trials of supervised injecting facilities in locations where homelessness and street-based injection are common [7].

## Abbreviation

AHR: adjusted hazard ratio
